# Supplementary material for: Systemic Inflammation Modulates Clearance and Drives Extra‐Hepatic Distribution of Extracellular Vesicles
Source: J Extracell Vesicles. 2026 Apr 7;15(4):e70256. doi: 10.1002/jev2.70256 (PMC13054838; doi:10.1002/jev2.70256)
Supplement: Supplementary file 2 — Supporting Information: jev270256‐sup‐0002‐Supplementary Table 1.docx [file JEV2-15-e70256-s001.docx]

**Supplementary Table**

| **Protein.Group** | **Protein.Names** | **Genes** |
| --- | --- | --- |
| P62897;cRAP-P99999 | CYC_MOUSE;cRAP-CYC_HUMAN | Cycs;cRAP-CYCS |
| P62983;cRAP-P62979 | RS27A_MOUSE;cRAP-RS27A_HUMAN | Rps27a;cRAP-RPS27A |
| Q61411;cRAP-P01112 | RASH_MOUSE;cRAP-RASH_HUMAN | Hras;cRAP-HRAS |
| cRAP-O00762 | cRAP-UBE2C_HUMAN | cRAP-UBE2C |
| cRAP-O43790 | cRAP-KRT86_HUMAN | cRAP-KRT86 |
| cRAP-O76011 | cRAP-KRT34_HUMAN | cRAP-KRT34 |
| cRAP-O82803 | cRAP-SRPP_HEVBR | cRAP-SRPP |
| cRAP-P00366 | cRAP-DHE3_BOVIN | cRAP-GLUD1 |
| cRAP-P00698 | cRAP-LYSC_CHICK | cRAP-LYZ |
| cRAP-P00761 | cRAP-TRYP_PIG |  |
| cRAP-P00918 | cRAP-CAH2_HUMAN | cRAP-CA2 |
| cRAP-P00921 | cRAP-CAH2_BOVIN | cRAP-CA2 |
| cRAP-P01008 | cRAP-ANT3_HUMAN | cRAP-SERPINC1 |
| cRAP-P01031 | cRAP-CO5_HUMAN | cRAP-C5 |
| cRAP-P02662 | cRAP-CASA1_BOVIN | cRAP-CSN1S1 |
| cRAP-P02753 | cRAP-RET4_HUMAN | cRAP-RBP4 |
| cRAP-P02768 | cRAP-ALBU_HUMAN | cRAP-ALB |
| cRAP-P02769 | cRAP-ALBU_BOVIN | cRAP-ALB |
| cRAP-P02787 | cRAP-TRFE_HUMAN | cRAP-TF |
| cRAP-P02788 | cRAP-TRFL_HUMAN | cRAP-LTF |
| cRAP-P04040 | cRAP-CATA_HUMAN | cRAP-CAT |
| cRAP-P04264 | cRAP-K2C1_HUMAN | cRAP-KRT1 |
| cRAP-P04745 | cRAP-AMY1_HUMAN | cRAP-AMY1A |
| cRAP-P06396 | cRAP-GELS_HUMAN | cRAP-GSN |
| cRAP-P07339 | cRAP-CATD_HUMAN | cRAP-CTSD |
| cRAP-P08758 | cRAP-ANXA5_HUMAN | cRAP-ANXA5 |
| cRAP-P09211 | cRAP-GSTP1_HUMAN | cRAP-GSTP1 |
| cRAP-P10599 | cRAP-THIO_HUMAN | cRAP-TXN |
| cRAP-P12081 | cRAP-SYHC_HUMAN | cRAP-HARS |
| cRAP-P12763 | cRAP-FETUA_BOVIN | cRAP-AHSG |
| cRAP-P13645 | cRAP-K1C10_HUMAN | cRAP-KRT10 |
| cRAP-P15252 | cRAP-REF_HEVBR |  |
| cRAP-P22629 | cRAP-SAV_STRAV |  |
| cRAP-P35527 | cRAP-K1C9_HUMAN | cRAP-KRT9 |
| cRAP-P35908 | cRAP-K22E_HUMAN | cRAP-KRT2 |
| cRAP-P61626 | cRAP-LYSC_HUMAN | cRAP-LYZ |
| cRAP-P61769 | cRAP-B2MG_HUMAN | cRAP-B2M |
| cRAP-P62937 | cRAP-PPIA_HUMAN | cRAP-PPIA |
| cRAP-P68871 | cRAP-HBB_HUMAN | cRAP-HBB |
| cRAP-P69905 | cRAP-HBA_HUMAN | cRAP-HBA1 |
| cRAP-Q06830 | cRAP-PRDX1_HUMAN | cRAP-PRDX1 |
| cRAP-Q15323 | cRAP-K1H1_HUMAN | cRAP-KRT31 |
| cRAP-Q58D62 | cRAP-FETUB_BOVIN | cRAP-FETUB |
| cRAP-rLys-C | cRAP-rLys-C | cRAP-rLys-C |

**Supplementary Table 1:** List of proteins identified as common contaminants (cRAP) and red blood cell (RBC)–associated proteins that were excluded from the proximity labeling dataset during analysis of the extracellular vesicle (EV) protein corona.
